# Multiscale Modeling of Epoxy-Based Nanocomposites Reinforced with Functionalized and Non-Functionalized Graphene Nanoplatelets

**DOI:** 10.3390/polym13121958

**Published:** 2021-06-13

**Authors:** Hashim Al Mahmud, Matthew S. Radue, Sorayot Chinkanjanarot, Gregory M. Odegard

**Affiliations:** 1 Department of Mechanical Engineering, University of Kufa, P.O. Box 21, Kufa, Najaf Governorate, Iraq; hashimn.almahmood@uokufa.edu.iq or; 2 Department of Mechanical Engineering—Engineering Mechanics, Michigan Technological University, Houghton, MI 49931, USA; msradue@mtu.edu; 3 National Metal and Materials Technology Center (MTEC), National Science and Technology Development Agency (NSTDA), 114 Thailand Science Park, Thanon Pahonyothin, Tambon Khlong Nueng, Amphoe Khlong Luang, Pathum Thani 12120, Thailand; sorayot.chi@mtec.or.th

**Keywords:** MD modeling, ReaxFF, functionalization, interphase/interface region, nanoplatelet content, nanoplatelet wrinkling, nanoplatelet aspect ratio

## Abstract

The impact on the mechanical properties of an epoxy resin reinforced with pristine graphene nanoplatelets (GNP), highly concentrated graphene oxide (GO), and functionalized graphene oxide (FGO) has been investigated in this study. Molecular dynamics (MD) using a reactive force field (ReaxFF) has been employed in predicting the effective mechanical properties of the interphase region of the three nanocomposite materials at the nanoscale level. A systematic computational approach to simulate the reinforcing nanoplatelets and probe their influence on the mechanical properties of the epoxy matrix is established. The modeling results indicate a significant degradation of the in-plane elastic Young’s (decreased by ~89%) and shear (decreased by ~72.5%) moduli of the nanocomposite when introducing large amounts of oxygen and functional groups to the robust sp^2^ structure of the GNP. However, the wrinkled morphology of GO and FGO improves the nanoplatelet-matrix interlocking mechanism, which produces a significant improvement in the out-of-plane shear modulus (increased by 2 orders of magnitudes). The influence of the nanoplatelet content and aspect ratio on the mechanical response of the nanocomposites has also been determined in this study. Generally, the predicted mechanical response of the bulk nanocomposite materials demonstrates an improvement with increasing nanoplatelet content and aspect ratio. The results show good agreement with experimental data available from the literature.

## 1. Introduction

The success of using advanced composite materials as an alternative for traditional metallic alloys represents a great milestone in the aerospace industry [1,2,3]. However, there is a need to continue developing new composites and nanocomposites to meet increasing demands on mechanical performance of aerospace composite structures. For that, the natural affinity between carbon allotropes and epoxy monomers, which can be chemically improved, has been utilized to promote the reinforcement/matrix interfacial adhesion [4,5,6,7]. The integrity of nanofillers and their content, shape, size, orientation, dispersion, and interaction with the epoxy matrix play an important role in the properties of nanocomposites [8,9,10,11,12]. As a result, an improved load transfer between the reinforcement and matrix can be achieved by optimizing these parameters.

The one-atom-thick and defect-free sheet of graphene, which was discovered in 2004, is the strongest ever tested material [13,14]. The highly specific surface area, unique graphitized plane structure, and high charge mobility of graphene have been harnessed in improving the mechanical/thermal/electrical properties of polymers. The exceptional thermo-mechanical properties of graphene nanoplatelets makes them excellent candidates for strengthening the epoxy matrix phase of composites [15]. Among the early experimental studies, Stankovitch et al. [16] and Ramanathan et al. [17] established that the homogeneous dispersion of large surface area and chemically modified graphene nanoplatelets in organic polymers can lead to a remarkable improvement in polymer engineering properties. In most of these experimental studies [17,18,19,20], the inclusion of a small amount (0.1–5.0 wt%) of graphene nanoplatelets has significantly improved the mechanical properties of polymer resins.

While the graphene nanoplatelet waviness/wrinkling is found to enhance the interlocking mechanism with the polymer matrix, graphene nanoplatelet agglomeration, which is triggered by the noncovalent van der Waals (vdW) force and the π-conjugation effect, is found to hinder graphene nanoplatelet dispersion in the polymer matrix [16,21,22,23,24]. Poor graphene nanoplatelet dispersion can significantly limit the reinforcing function of graphene nanoplatelets by reducing the interfacial contact surface area and providing easy slip planes within the reinforcement [25]. In addition to rigorous mixing and stirring techniques, chemical modification of graphene nanoplatelet surfaces (functionalization) has been widely used for improving graphene nanoplatelet dispersion and adhesion with polymers [16,26,27]. Even though chemical modification alters the strong sp^2^ hexagonal structure into a weaker sp^3^ structure, experimental studies have shown that it can improve the mechanical and other engineering properties of the produced nanocomposite [28,29,30,31,32,33,34]. This improvement is highly governed by the functional group type, molecular configuration, and concentration [35,36,37,38]. Despite the limited number of studies dedicated to investigating the effect of graphene nanoplatelet functionalization, there is a need for a systematic approach to understanding the role of different types of graphene nanoplatelet modification and functionalization on the resulting composite mechanical properties.

The aspect ratio of the filler used to reinforce the polymer matrix represents another essential factor that can greatly affect the properties of the composite. For polymer matrix reinforced with microscale fibers; such as carbon fibers (CF) and fiberglass, an infinite aspect ratio is commonly used for the fiber unless it is chopped. However, for the nanoscale fillers or particles, such as graphene nanoplatelets and carbon nanotubes, the situation is different as these nanofillers are produced with a wide range of aspect ratios [39,40,41,42]. The reported results from experiments that have been conducted to capture the influence for particular carbon nanofiller sizes (aspect ratio) mostly refer to an improvement in the engineering properties of nanocomposites as the aspect ratio increases [41,42,43,44]. Unfortunately, experimental techniques are infeasible and limited to investigating the role of aspect ratio in reinforcing polymer matrix with nanoscale fillers. Therefore, there is a need to fully understand the effect of changing the aspect ratio on polymer matrix properties.

Although there are a wide range of experimental characterization tools that provide information about the structure and properties of nanocomposites, there is information that they cannot easily provide. For example, intermolecular interaction energy, interphase region characteristics, and molecular-level mechanical failure behavior cannot be fully resolved utilizing the available experimental tools. For this reason, computational simulation techniques have been widely integrated with experiments to provide a more comprehensive understanding of material structure and behavior at the atomic scale. Molecular dynamics (MD) simulation is one of the most powerful computational tools that can be utilized to predict properties of different types of materials. Polymers and their nanocomposite materials have been broadly investigated with MD modeling to predict molecular structure and physical behavior under different types of mechanical loadings and thermal conditions [45,46,47,48].

As MD modeling simulations can be computationally expensive for studying multiple case studies considering the wide range of composite material parameters, fast and efficient microscale computational approaches represent the typical tools for this purpose [49,50]. MD modeling combined with micromechanics has been used in computational studies to predict the mechanical properties of randomly dispersed graphene nanoplatelets in epoxy polymers [51,52,53,54]. Different graphene nanoplatelets content, dispersion levels, and chemical modifications of the graphene nanoplatelet surface can be considered. The variety of functional groups that can be introduced to the graphene nanoplatelet surface opens the door for more options for tailoring the engineering properties of the composite material. The modified graphene nanoplatelets show higher dispersion levels and better mutual interaction with the hosting matrix.

Due to the limitations in computational resources used for MD modeling, computational studies are usually constrained to a few nanoseconds for type MD models of organic materials (a few thousand atoms). This could lead to significant uncertainty in the predicted material properties due to the small statistical sampling of the molecular structure and the presence of significant thermal fluctuations at non-zero temperatures. The use of multiple model samples (replicates) for MD simulation can help to capture the variance in predicted properties due to these factors. The force field selection is another critical factor used for accurate predictions in MD modeling. As MD modeling with a reactive force field requires significant simulation times, most computational studies have been constrained to the use of fixed-bond force fields, which are less computationally expensive. However, reactive force fields have been shown to accurately predict mechanical properties of epoxy-based materials [47,48,55].

The aim of this study is to develop a material-designing computational approach for graphene nanoplatelet/epoxy nanocomposites. Various graphene nanoplatelet types are considered: pristine (GNP), functionalized with oxygen groups (GO), and further functionalized with amine groups (FGO). The adopted approach involves a multiscale analysis procedure to relate molecular structure to bulk level mechanical properties of the composite materials. For the nanoscale level, MD modeling is used to establish the molecular structure and load-transfer capability of the interphase region between GNP, GO, or FGO and epoxy. The load transfer is quantified using interfacial interaction energy, interfacial adhesion, nanoplatelet wrinkling, and mechanical interlocking. A reactive force field is used to more accurately simulate the nonlinear force-distance relationship (and potential scission) of covalent bonds. At the micro and macro-levels, micromechanics is used to predict the effect of the nanofiller functionalization, content, and aspect ratio on the mechanical response of the nanocomposite material. The outcomes of the modeling approach were compared with several experimental studies in the literature.

## 2. Molecular Dynamics Modeling

For the nanoscale computational work, MD simulations were performed using the open-source LAMMPS software package (Sandia National Laboratories, Albuquerque, NM) [56,57]. The MD modeling procedure adopted in this study is based on a procedure published previously [55]. The MD models were initially built using a fixed-bond force field (OPLS) [58,59] to perform crosslinking reactions of epoxy monomers. A transition simulation into an efficient reactive force field (ReaxFF) with the parameterization of Liu et al. [60] was subsequently performed to all MD models, followed by equilibration to prepared for mechanical property prediction at the nanoscale level. Three-dimensional periodicity was considered for all MD models to mimic the bulk behavior of the material. Visual molecular snapshots presented herein were rendered using the open source OVITO software package [61].

### 2.1. Nanocomposite Constituents

Three unique nanocomposites were considered in this study to address the functionalization effect on the mechanical properties of the material. Simulated epoxy unit cells reinforced with GNP, GO, or FGO were created using LAMMPS. The size of each MD model was carefully assigned to only capture the graphene and surrounding epoxy interphase, not the bulk epoxy, as discussed previously [54,55]. Five MD replicates for each nanocomposite sample were created to explore the possible variation in the obtained mechanical properties from each case study.

#### 2.1.1. Epoxy Monomers

**Figure 1:**
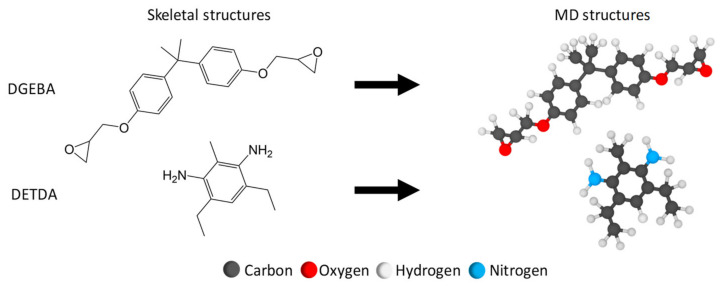
Molecular structures of epoxy monomers.

EPON 828 (diglycidyl ether of bisphenol A, DGEBA) as the epoxy resin, and the EPIKURE curing Agent W (diethyltoluenediamine, DETDA) as the hardener, were simulated as the epoxy matrix in this study. One EPON 828 molecule (49 atoms) and one DETDA molecule (31 atoms) were individually created (Figure 1) via the ChemDraw Professional software package, PerkinElmer Informatics, Inc. The molecular structure was initially established using the OPLS-All Atom (OPLS-AA) force field with a stoichiometric mixture ratio of two DGEBA molecules to one DETDA molecule. A single unit of the stoichiometric mixture (2:1) was simulated in a periodic MD box totaling 129 atoms. The mixture was replicated 48 times to form a larger system comprising 144 monomers (96:48) with 6192 atoms.

#### 2.1.2. Graphene Nanoplatelets (GNP, GO, and FGO)

**Figure 2:**
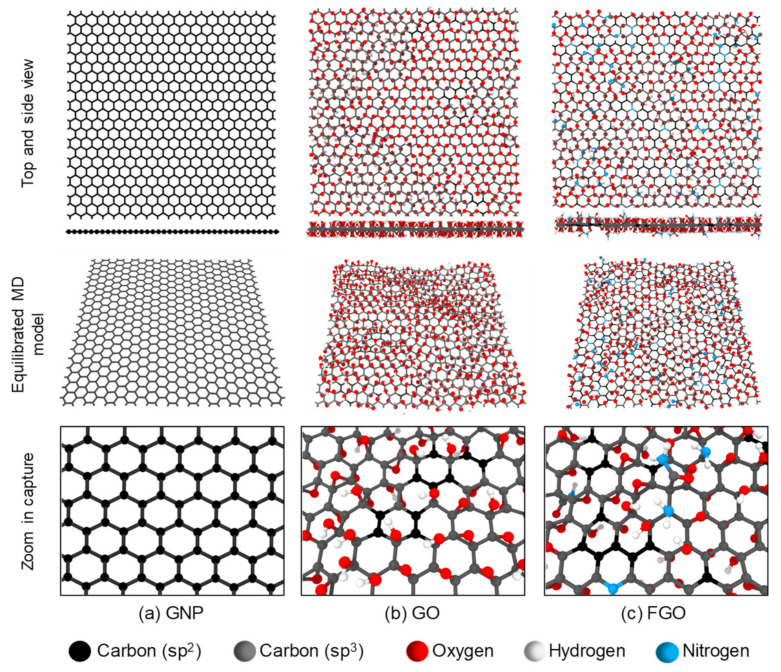
Representative MD models of GNP, GO, and FGO nanoplatelets (**a**–**c**).

The “lattice” command was used in a LAMMPS script to create a single layer of GNP. The lattice parameters for the hexagonal atomic structure of a pristine sheet of graphene were taken from Gray et al. [62]. A square-GNP-layer was created that includes 836 carbon atoms with lateral dimensions of ~47.5 Å along the *x*-axis and ~47.7 Å along *y*-axis, as shown in Figure 2a. For the GO and FGO nanoplatelets, the functional groups were randomly attached to the top and bottom surfaces of the GNP layer. The chemical composition and quantitative data for GO and aminated GO (which is henceforth referred to as FGO) were taken from the surface element analysis results of X-ray photoelectron spectroscopy (XPS) performed by Park et al. [34]. Note the FGO represents GO nanoplatelets treated with ammonia solution of 21% concentration as reported by the experimental study. Table 1 includes the elemental contents (atomic percent: at%) from which the GNP and functional groups atomic data were derived and that were used in simulating GO and FGO nanoplatelets.

For the GO MD model, the core-level spectra of the oxygen content (Ols = 39.35 at%) was assumed to be 50% of epoxide (–O–) and 50% of hydroxyl (–OH) functional groups as there is no detailed information about the chemical composition of the oxygens in the reported data. It is important to note that each oxygen group has a unique effect on the GNP structure as each epoxide oxygen atom forms two covalent bonds with two carbon atoms in the GNP, while each hydroxyl oxygen atom forms only one covalent bond with GNP. This implies that epoxide groups have a greater detrimental effect on the robust structure of the GNP by turning a greater number of sp^2^ bonds into the weaker sp^3^ structure. In other words, the sp^3^/sp^2^ bond ratio (calculated using a Python script) in the GNP oxidized with a specific amount of epoxide groups is double of that if the oxygens were presented in the hydroxyl form. Generally, the interfacial interaction is highly dependent on the functional group size, chemical composition, and molecular configuration [63,64]. Figure 2b shows a representative MD model of GO created according to the data presented in Table 1 using the Lerf-Klinowski GO model [65].

For the FGO MD model, the core-level spectra of the oxygen content (Ols = 31.67 at%) was assumed to be 50% epoxide (–O–) and 50% hydroxyl (-OH) functional groups. The core-level spectra of the nitrogen content (Nls = 4.35 at%) was assumed to be 59.1% amine groups (–NH_2_), 20.8% amide groups (O=C–NH_2_), and 20.1% graphitic nitrogen (–N–). These values were taken from curve fitting of the Nls spectra of aminated A21-GO performed by Park et al. [34]. It is important to note that due to the lack of information regarding the chemical form of nitrogen content in the experimental data, and for modelling simplicity, all the nitrogen content in the form (–N–) were simulated as graphitic nitrogen, which does not damage the hexagonal carbon rings in the GNP. The nitrogen doping process involved taking out 11 carbon atoms from the GNP lattice and replacing them with 11 nitrogen atoms. Regarding the other forms of nitrogen, however, both Pyrrolic-N and Pyridinic-N as the other types of nitrogen-doped graphene were found to cause structural defects (voids) in the graphene lattice. While Pyrrolic-N was found to exist within pentagon rings in the graphene lattice [66], Pyrazole-N was found to exist at the edges of the graphene lattice [67]. Figure 2c shows a representative MD model of FGO created according to the data presented in Table 1.

All nanoplatelets (GNP, GO, and FGO) were simulated with continuous lateral edges (periodic boundary conditions). The presence of carboxyl and carbonyl as functional groups in GO and FGO was ignored because they are more likely to exist in low concentrations at the edges and in defected regions (open rings or voids) in the graphene nanoplatelets [8,16]. Five MD replicates for each nanoplatelet type (GNP, GO, and FGO) were simulated to account for the possible variation in the predicted properties. Each MD replicate of the GO and FGO had a unique random distribution of the attached functional groups. However, the chemical concentration and content of functional groups were preserved for all GO and FGO MD replicates.

Both GO and FGO MD models shown in Figure 2b,c exhibit a degree of wrinkling. This structural feature is mainly attributed to the presence of the functional groups on the graphene nanoplatelet surface [17,29]. However, pristine GNP with a relatively large aspect ratio can also have a wrinkled structure [42]. Introducing functional groups onto the planar/unwrinkled structure of the graphene nanoplatelets shown in Figure 2a can greatly diminish its robust sp^2^ structure. The functionalization degree can be used to specify the sp^3^/sp^2^ ratio, which is a measure of the graphene nanoplatelet purity and its structural robustness. The lower the sp^3^/sp^2^ ratio, the more pristine and planar the graphene nanoplatelets are. Table 1 includes the sp^3^/sp^2^ ratio for the simulated nanoplatelets. While pristine GNP has a fully intact sp^2^ structure, GO and FGO exhibit sp^3^/sp^2^ of 0.973 and 0.798, respectively. This indicates that GO has the highest functionalization degree; however, both GO and FGO are almost completely covered with functional groups.

**Figure 3:**
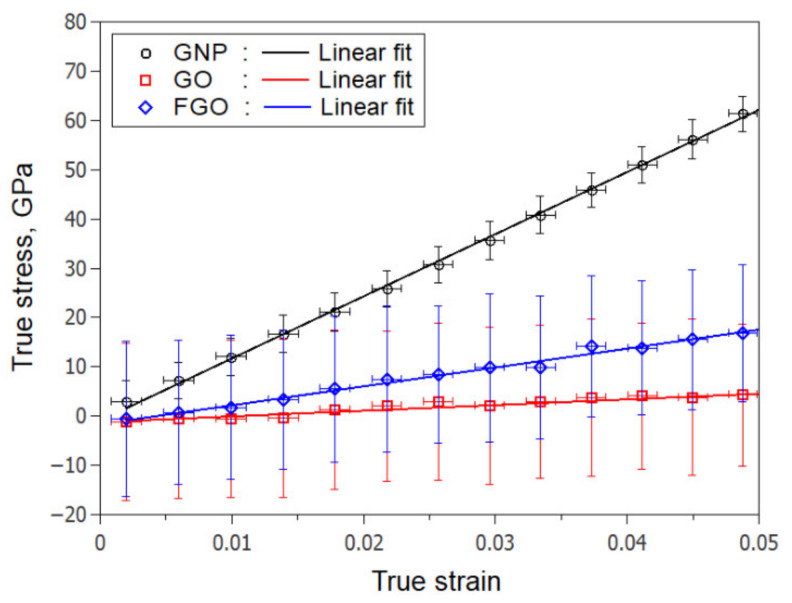
Representative uniaxial stress-strain response for GNP, GO, and FGO nanoplatelets.

To determine the effect of functionalization on the graphene nanoplatelet structural integrity, the three nanoplatelet types were subjected to simulated uniaxial tensile deformations along the zigzag-axis. Figure 3 shows representative stress-strain responses of GNP, GO, and FGO. Clearly, the GNP response exhibits the highest elastic modulus of 1264 GPa. Both FGO and GO exhibit significantly lower moduli of 386 GPa and 119 GPa, respectively. Consequently, the nanoplatelet stiffness can be arranged according to their elastic moduli in the order: GNP ≫ FGO > GO. That is, the lower the sp3/sp2 or functionalization degree, the stiffer the graphene nanoplatelets. Even though the presence of excessive functional groups degrades the stiffness of the functionalized graphene nanoplatelets, evidence that functionalization can produce some other good features for the molecular structure of the nanocomposite is provided below.

### 2.2. Nanoplatelet Dispersion (d-Spacing)

**Figure 4:**
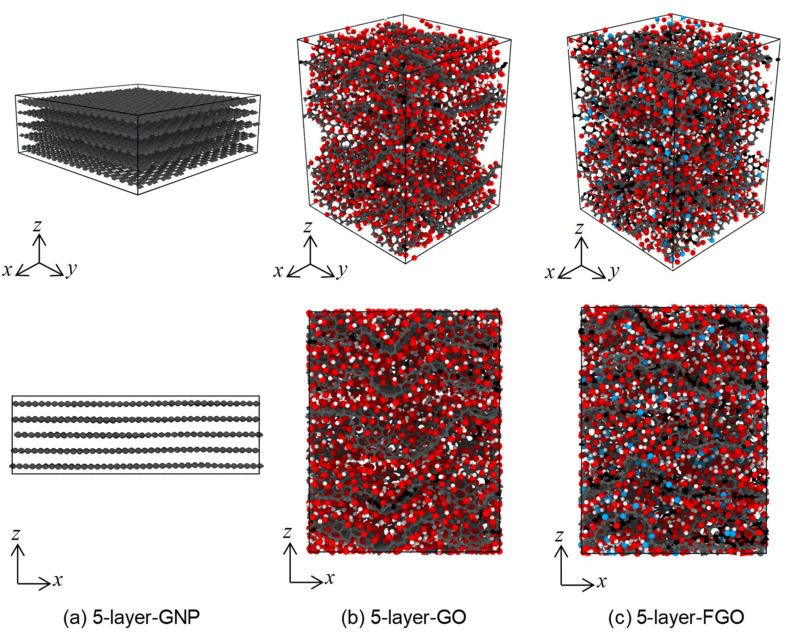
Equilibrated MD models of GNP, GO, and FGO stacked nanoplatelets (**a**–**c**).

To understand how the functional groups can help in improving the graphene nanoplatelet dispersion, the following MD simulations were performed. The modeling procedure is inspired by the Hummers method [68] for large production of GO nanoplatelets from bulk graphite. In this method, the carbon-to-oxygen atomic ratio of the produced GO nanoplatelets was reported to be 2.1–2.9. The MD simulations involved mimicking the process of preparing GO and FGO from pristine GNP. Three MD models were simulated, where each model consisted of five unique layers of GNP, GO, and FGO stacked together. Figure 4 shows the equilibrated MD models using ReaxFF with simulation box dimensions: (47.7)x Å × (47.8)y Å × (16.5)z Å for 5-layer-GNP MD model, (42.0)x Å × (42.5)y Å × (54.3)z Å for 5-layer-GO MD model, and (42.2)x Å × (41.1)y Å × (57.0)z Å for 5-layer-FGO MD model. Clearly, introducing the oxygen and functional groups to the bulk GNP resulted in an increase in the overall simulation box volume by 157.6% and 162.8% for GO and FGO, respectively. These volume increments are attributed to the increase in the size along the z-axis that surpasses the reduction in the lateral sides caused by wrinkling in GO and FGO nanoplatelets. Table 2 includes the predicted distance between individual nanoplatelets (d-spacing) compared with experimental observation values from the literature. The predicted d-spacing was calculated using two approaches: first, by roughly dividing the MD simulation box length along the z-axis (lz) by the number of the nanoplatelets (5 layers); and second, by obtaining the mean value of the measured distance between the center of mass for every two neighboring nanoplatelets. Generally, the predicted d-spacing values between GNP, GO, and FGO stacked nanoplatelets using both approaches agree with the experimental observations. The slight discrepancy in the predicted d-spacing for GO and FGO stacked nanoplatelets with the experimental observation values can be attributed to the amount of oxygen and the other functional groups presented in each case. It has been reported that the d-spacing in graphite oxide increases as the humidity level increases, which results in various d-spacing values ranging from 6 to 12 Å between the graphene nanoplatelets [69].

The interaction energy between the stacked nanoplatelets was also investigated for the three material models. The interlayer interaction energy for each layer with the other four layers in the same sample was calculated by subtracting the isolated potential energy of the selected four layers from the overall potential energy including the fifth layer. The mean value of the interlayer interaction energy was determined over the five layers in each sample. It is important to note that the higher the negative value of the interlayer interaction energy, the stronger the interaction between the nanoplatelets. The mean interlayer interaction energies for the 5-layer-GNP, 5-layer-GO, and 5-layer-FGO MD models are 7456 ± 15, 6348 ± 80, and 6860 ± 148 kcal/mol, respectively. Clearly, the interlayer interaction energy decreases with the introduction of oxygen groups to the GNP. However, replacing some of the oxygen groups with amine/amide functional groups restores some of the interlayer interaction energy. This can be attributed to the change in the pi-stacking between GNP layers, which depends on the sp^2^ structural integrity (refer to Table 1).

Consequently, the increase in the d-spacing value with the relatively low interlayer interaction energy can facilitate the separation of GO and FGO layers using mild sonication in water or organic solvent mediums. The process of preparing nanocomposites involves three essential steps: first, the mixing of the filler nanoplatelets with the polymer resin under a stirring action; second, the constituents’ interaction, which involves the formation of covalent bonds between functional groups and the resin; and third, an overall curing of the polymer network, which is accomplished with the aid of a curing agent (hardener) and temperature [16,25,26,27]. These three steps can be effective in alleviating or even preventing the agglomeration of nanoplatelets. Based on the predicted interfacial interaction energy values, it can be inferred that the restacking phenomena in GO and FGO is lower than that in GNP. Thus, GO and FGO nanoplatelets can provide better dispersion levels in polymer matrices in comparison to GNP.

### 2.3. Nanocomposite MD Models

The MD models of the unpolymerized and low-density epoxy monomers were combined with GNP, GO, and FGO MD models to form MD unit cells of the nanocomposites. The lateral size of each MD unit cell was governed by the equilibrated lateral size of the nanoplatelets. As five unique MD replicates of each nanoplatelet were already created, five MD replicates of GNP/, GO/, and FGO/epoxy nanocomposite unit cells were consequently created. The total number of atoms in the GNP/, GO/, and FGO/epoxy MD models were 7028, 7841, and 7811 atoms, respectively. This combining process involved MD simulation settings to maintain the center of mass (COM) of the nanoplatelets at the center of the simulation box. In addition, each MD model was subjected to slow densification (size reduction) simulation steps for 2.5 ns along the normal axis to the plane of the nanoplatelets to densify the epoxy monomers to the bulk level density (~1.2 g/cm^3^). Each densifying simulation had a unique velocity seed number to maintain a random and unique distribution of the epoxy monomers for each MD replicate. All the densifying simulations were performed with the NPT (constant number of atoms, pressure, and temperature) ensemble with the Nose/Hoover barostat along the lateral direction at 300 K. All lateral surfaces (normal to the x- and y-axes) of MD simulation boxes were maintained at 1 atm pressure. Hence, the nanoplatelets were free to adjust their lateral size under the normal compression from the epoxy monomers. To maintain a thermodynamically equilibrated molecular structure once the densification was complete, the systems were subjected to 1 ns of MD simulation with the NVT (constant number of atoms, volume, and temperature) ensemble followed by a molecular minimization (MM) simulation. The simulations involved the use of the Nose/Hoover thermostat with a temperature ramped up to 600 K, then ramped down to 300 K.

The epoxy monomers in the nanocomposite MD models were subjected to crosslinking simulations utilizing an approach used in a previous work [55]. The polymerization process of epoxy monomers in all MD models was performed to about an 80% crosslinking density (the fraction of the formed crosslinked to all potential crosslink sites). In order to mimic the actual functionalized nanocomposite synthesis process mentioned above, the computational crosslinking was first randomly performed between the epoxide reactive radicals in the DGEBA monomers and the amine/amide functional groups on the FGO nanoplatelet. That is, covalent bonding between the FGO and the epoxy network was first established. This step of crosslinking was stabilized at an overall 20% crosslink density. The next step of crosslinking was performed for the epoxy monomers, which increased the overall crosslink density by an additional 60%.

Once the polymerization simulations were completed, all MD models were further equilibrated for 1 ns with 1 fs time steps. The equilibration simulations were performed using the NPT ensemble at 300 K with the Nose/Hoover anisotropic barostat to minimize residual stresses produced during the crosslinking process and to stabilize the molecular structure. Transition simulations from OPLS to a reactive force field (ReaxFF) with the parameterization of Liu et al. [60] were performed for the equilibrated MD models. ReaxFF has been validated for simulating epoxy systems and their nanocomposites [47,48,50,55]. The transition process was performed gradually over 1 ns using 0.1 fs time steps with the temperature progressively ramping up from 1 K to 300 K. The low temperature was required to limit the natural vibrations and velocities of the atoms at the beginning of the transition simulation. These MD simulation settings were found to help in maintaining the integrity of molecular structure during the transition process [55]. The MD models were then all equilibrated with ReaxFF for 1 ns using 0.1 fs time steps. The equilibration simulations involved the use of the NPT ensemble at 300 K with the anisotropic Nose/Hoover barostat followed by MM simulations. It is important to note that all the nanoscale analyses and predictions were performed on these well-equilibrated MD models with ReaxFF.

**Figure 5:**
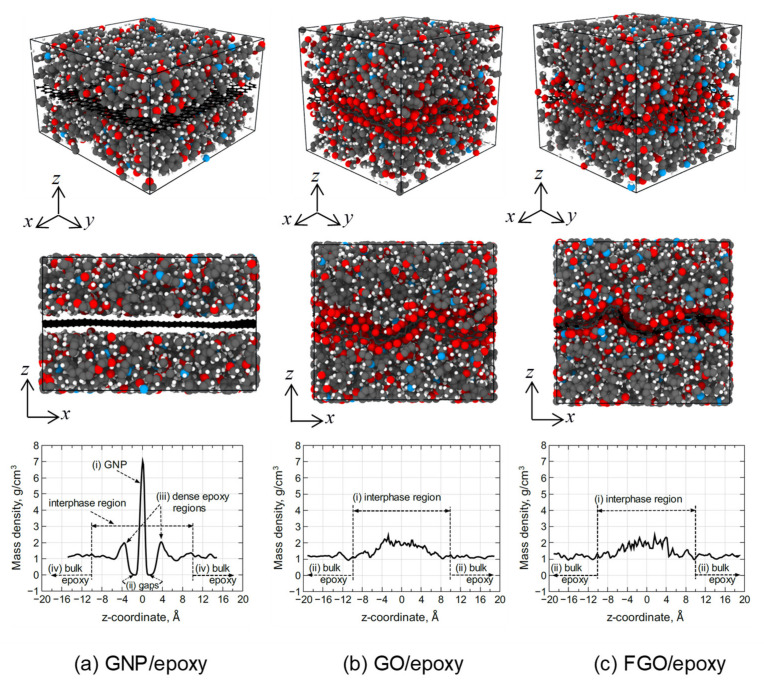
Representative MD models of the nanocomposites with their mass density distribution along z-coordinate (**a**–**c**).

Figure 5 shows representative MD models of the GNP/, GO/, and FGO/epoxy nanocomposites with the mass density distribution along the *z*-axis. The COM point of each nanocomposite MD model was selected at the origin (0,0,0) of the *x*, *y*, and *z* coordinates. The average simulation box size of the GNP/epoxy MD models was (47.55 ± 0.03)*_x_* Å × (47.67 ± 0.04)*_y_* Å × (29.68 ± 0.30)*_z_* Å. Likewise, the average simulation box size of the GO/epoxy MD model was (42.38 ± 1.97)*_x_* Å × (43.41 ± 0.83)*_y_* Å × (38.42 ± 2.35)*_z_* Å, and the average size of the FGO/epoxy models was (43.39 ± 1.16)*_x_* Å × (42.42 ± 0.83)*_y_* Å × (38.51 ± 1.16)*_z_* Å. For all three nanocomposite types, the model size was sufficiently large enough to capture the entire interphase region, which was previously shown to be 10 Å wide [55]. It is noteworthy to mention that the size of the simulation box is governed by the lateral size of the nanoplatelets. The wrinkling effect in GO and FGO nanoplatelets resulted in a decrease in the lateral dimensions of their composite simulation boxes relative to the flat GNP/epoxy composite.

The mass density distribution of the GNP/epoxy MD model exhibits a clearly defined profile reflected about the COM. There are four distinct regions that can be recognized from the mass density profile of the GNP/epoxy MD model: (i) the large spike at the center, which represents the flat GNP atoms; (ii) the gap region between the GNP and the epoxy, which is caused by the repulsive portion of the van der Waals forces exerted by the GNP and epoxy atoms [50]; (iii) the small spike next to the gap, which represents a dense epoxy region (~2 g/cm^3^ at 4 Å from the GNP) followed by a density drop to ~1 g/cm^3^ and a gradual rise to the bulk density at ~10 Å from center (it has been reported previously that most of the phenyl rings in the epoxy network were observed to be aligned with GNP surface at this region [55]); and (iv) the bulk epoxy region (~1.2 g/cm^3^) in which the interfacial interaction between GNP and epoxy diminishes with distance from the GNP.

For the GO/epoxy and FGO/epoxy nanocomposite models, two distinct regions can be recognized from their mass density profiles: (i) the interphase region, which includes the diffuse nanoplatelets (GO or FGO), and the adjacent highly interacted epoxy region with a density of ~2 g/cm^3^ along z (−4:4 Å), which decreases with distance to the bulk density at ~10 Å from center; and (ii) the bulk epoxy region (~1.2 g/cm^3^) in which the interfacial interaction between the nanoplatelet and epoxy diminishes with distance away from GO or FGO nanoplatelets. According to the density profile analysis and MD models shown in Figure 5, it can be inferred that both GO and FGO provide better attraction with the epoxy matrix than GNP, as no gaps were observed in the interfacial region. In addition, the wrinkling and surface roughness in GO and FGO are expected to improve the interlocking mechanism with the epoxy matrix, which will be discussed in more detail below.

### 2.4. Waviness Factor

In most experimental observations, graphene nanoplatelets exhibit one to several stacked-nanosheets with different sizes and morphologies having planar or wrinkled structures with sharp or folded edges [22,39,70]. The graphene nanoplatelet morphology can be governed by two primary factors. Graphene nanoplatelets produced with only a few graphene layers stacked together with relatively large aspect ratios can exhibit large amounts of waviness due to their relatively low bending stiffness. Additionally, the low integrity in the graphene nanoplatelet sp^2^ structure due to the potential presence of vacancies, defects, and functional groups can also increase the waviness degree [17,71]. It has been reported that a wrinkled graphene nanoplatelet surface can provide better interlocking mechanisms and strong interfacial interactions with the hosting matrix [20,72]. Therefore, studying the graphene nanoplatelet morphology is of great importance as it has been found to improve the mechanical properties of graphene-based nanocomposites.

The waviness (wrinkling) factor (WF) associated with graphene nanoplatelets has been defined as the ratio of the direct distance between the two ends of a wrinkled graphene nanoplatelet (wrinkled length, $L^{w}$) to the unwrinkled, unequilibrated graphene nanoplatelet (actual length, $L^{a}$) [71]. The WF of the graphene nanoplatelets within the epoxy matrix for all MD models simulated in this research was calculated. It is important to note that the original length of the GNP was considered to be as the lateral length (47.60 Å along *x*-axis, and 47.65 Å along *y*-axis) of an equilibrated planar GNP using ReaxFF. Table 3 includes the waviness factors along the *x*-axis (WF*_x_*) and *y*-axis (WF*_y_*) calculated for all replicates of GO/epoxy and FGO/epoxy MD models. The overall WF is calculated as the mean value of the averaged values over WF*_x_* and WF*_y_*. It is noteworthy to mention that all equilibrated GNPs exhibit an overall WF of ~1, which indicates a relatively flat GNP morphology in the GNP/epoxy MD models, as shown in Figure 5a. However, both GO and FGO exhibit an overall WF of (~0.9), which indicates a ~10% reduction in the lateral dimensions caused by the wrinkled morphology of the nanoplatelets, as shown in Figure 5b,c.

### 2.5. Weight and Volume Fractions of the Nanoplatelets

The properties of nanocomposites are typically evaluated with respect to the amount of the nanofiller used in reinforcing the polymer matrix. The nanofiller amount can be represented by either its weight fraction (wt%) or volume fraction (vol%). The typical goal of designing nanocomposites is to improve properties with the lowest amount of reinforcing nanofiller material. Excessive amounts of the nanofiller can make processing difficult (increased resin viscosity and filler agglomeration) and produce detrimental effects on the nanocomposite, such as increasing the weight and the number of critical micro-defects. Thus, it is necessary to study the nanocomposite properties at different levels of nanofiller reinforcement.

In this work, both wt% and vol% values were calculated for the GNP, GO, and FGO nanoplatelets in their nanocomposite MD models. For the wt% evaluation, a simple LAMMPS script was performed on each MD model to obtain the ratio of the partial weight of the nanoplatelet to the overall weight of the nanocomposite. The “group” command in LAMMPS was utilized to specify the atom types that were used to determine the partial weight for each nanoplatelet. For the vol% evaluation, an acceptable estimation can be made for the GNP utilizing the MD simulation box volume and its mass density distribution in Figure 5a since it is possible to estimate the planar GNP volume. However, this approach is not applicable with wrinkled GO and FGO nanoplatelets. Fortunately, the “compute voronoi/atom” LAMMPS command introduces an effective solution for estimating the vol% of each nanoplatelet despite its wrinkled topology. The calculation process is based on the 3D Voronoi tessellation method that was originally proposed by Gregory Voronoi (1908) [73].

**Figure 6:**
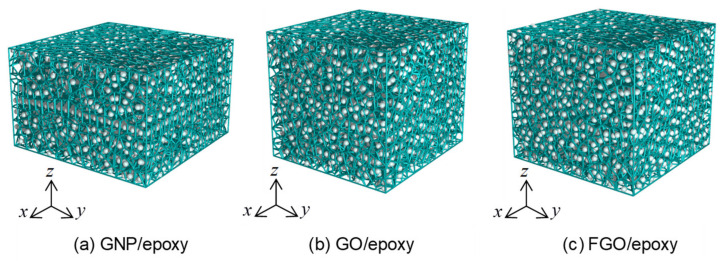
The 3D Voronoi tessellation of GNP/, GO/, and FGO/epoxy nanocomposite MD models; the green line mesh represents the Voronoi cells generated for each atom (white beads) (**a**–**c**).

Figure 6 shows representative samples of GNP/, GO/, and FGO/epoxy MD models analyzed using the 3D Voronoi tessellation. The green-wire-mesh was generated using the open source voro++ software library for the computation of the Voronoi tessellation [74], while the images were rendered using the Persistence of Vision Raytracer (POV-Ray) software package. Clearly, the overall volume for each MD model is the sum of the discretized volumes of the atoms in the system.

Table 4 includes the nanoplatelet wt% and vol% averaged over the five MD replicates of the nanocomposites. As the elemental content (at%) of each nanocomposite sample was unchanged over their MD replicates, the nanoplatelet wt% remains constant over the replicates. However, the different distributions of the oxygen and functional groups resulted in a slight variation in the nanoplatelet vol% over the MD replicates.

### 2.6. Nanoplatelet/Epoxy Interaction Energy

The interfacial interaction energy (IIE) can be used to assess the interfacial binding between nanoplatelets and epoxy matrix. The calculation of IIE involves subtracting the isolated potential energy of the nanoplatelet ($PE_{NP}$) and epoxy ($PE_{EPO}$) from the total potential energy of the entire model ($PE_{MD}$) [50,55]:

(1) $$IIE=PE_{MD}-PE_{NP}-PE_{EPO}$$

**Figure 7:**
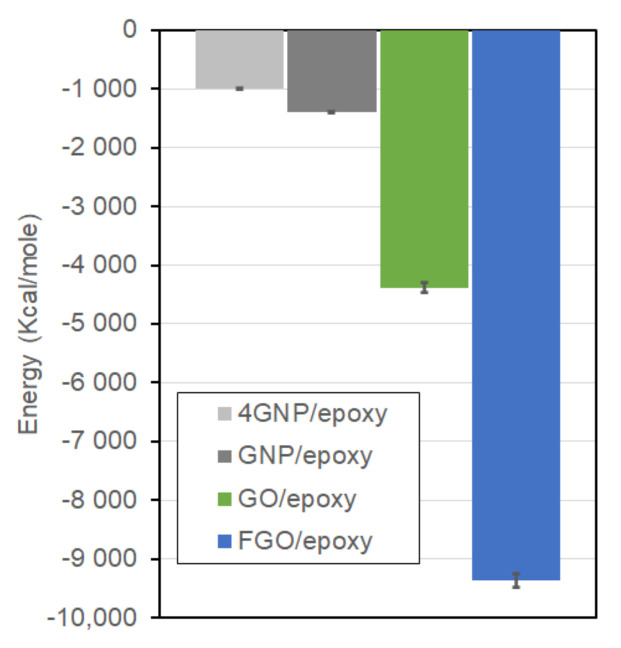
The interfacial interaction energy between epoxy matrix and 4GNP, GNP, GO, and FGO nanoplatelets.

Figure 7 shows the IIE levels evaluated for the current GNP/, GO/, and FGO/epoxy MD models in addition to the IIE of a 4-GNP-layer/epoxy (4GNP/epoxy) nanocomposite, which was modeled in a previous work [55]. The 4GNP/epoxy MD sample was modeled to account for the effect of GNP agglomeration on the predicted mechanical properties of the nanocomposite. It is important to note that the IIE for each nanocomposite type was averaged over the five MD replicates, which were well-equilibrated with ReaxFF. In addition, the larger the negative IIE magnitude, the stronger the nanoplatelet/epoxy interaction. Clearly, the FGO/epoxy MD model with −9356 kcal/mole exhibits the highest IIE among the other nanocomposites. The IIE of the GO/epoxy MD model with −4381 kcal/mole is second highest. However, both the GNP/epoxy and 4GNP/epoxy systems exhibit a much lower IIE of −1389 and −992 kcal/mole, respectively. Hence, the IIE of the FGO/epoxy nanocomposite surpasses the GO/, GNP/, and 4GNP/epoxy nanocomposites by 113.6%, 573.6%, and 843.2%, respectively. The IIE of GO/epoxy nanocomposite surpasses the GNP/epoxy and 4GNP/epoxy nanocomposites by 215.4% and 341.6%, respectively. The IIE of GNP/epoxy nanocomposite exceeds the IIE of 4GNP/epoxy nanocomposite by 40%, which can be attributed to the additional epoxy-epoxy interaction energy term across the single layer of GNP.

Accordingly, the presence of functional and/or oxygen groups on the GNP surface resulted in a significant improvement in the IIE with the hosting matrix. That is, the interaction between the GNP and epoxy matrix can be greatly enhanced by introducing functional groups to the GNP surface.

### 2.7. The Effective Mechanical Properties (MD Prediction)

To predict the effective mechanical properties, the MD models were subjected to six deformation MD simulations. As performed previously [55], each of the five MD replicates of GNP/, GO/, and FGO/epoxy nanocomposites were subjected to tensile strain simulations along the *x-* and *y*-directions to predict the in-plane elastic modulus $\left[E_{ip}=\left(E_{x}+E_{y}\right)/2\right]$ and in-plane Poisson’s ratio $\left[\nu_{ip}=\left(\nu_{xy}+\nu_{yx}\right)/2\right]$. Tensile strain simulations along the *z*-direction were used to predict the out-of-plane elastic modulus $\left[E_{op}=E_{z}\right]$ and out-of-plane Poisson’s ratio $\left[\nu_{op}=\left(\nu_{zx}+\nu_{zy}\right)/2\right]$. To predict the shear moduli, shear strain simulations in the *xy*-, *xz*-, and *yz*-planes were performed on each MD sample. While the deformation simulations in *xy*-plane were used to predict the in-plane shear modulus $\left[G_{ip}=G_{xy}\right]$, the deformation simulations in *xz*- and *yz*-planes were used to predict the out-of-plane shear modulus $\left[G_{op}=\left(G_{xz}+G_{yz}\right)/2\right]$. Consequently, 30 MD deformation simulations were performed on the replicates of each nanocomposite for a total of 90 MD deformation simulations for the three nanocomposite types. For the axial tensile strain simulations, the NPT ensemble at 300 K with the Nose/Hoover barostat along the lateral directions was utilized to maintain the lateral surfaces at 1 atm pressure. These simulation settings were imposed to account for the Poisson contractions. The shear strain simulations were performed with the NVT ensemble at 300 K. All the deformation simulations were carried out over a total simulation time of 0.5 ns using 0.1 fs time steps and a strain rate of 1 × 10^8^ s^−1^, which resulted in a maximum engineering strain of 5%.

Table 5 provides the predicted mechanical properties from the MD models. For comparison purposes, the predictions of a single-layer-GNP/epoxy from Hadden et al. [54] using the OPLS force field are included. The predictions of a 4-layer-GNP/epoxy (4GNP/epoxy) MD model from a previous work [55] are also included to explore the dispersion effect on the mechanical properties. The effective mechanical properties were averaged over the five MD replicates, thus providing the corresponding standard deviations as the prediction uncertainties.

## 3. Micromechanics Modeling

**Figure 8:**
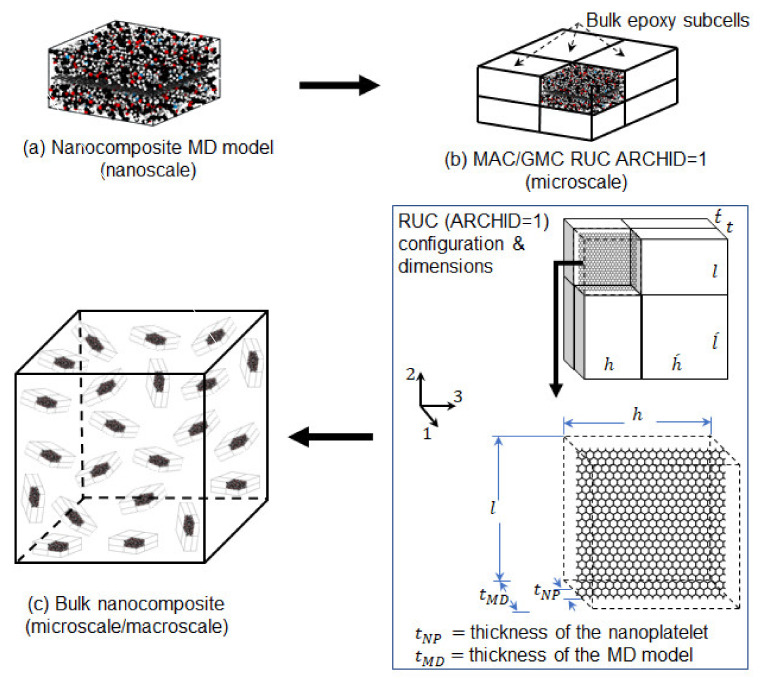
The multiscale modeling workflow (**a**–**c**).

The Micromechanics Analysis Code based on the Generalized Method of Cells (MAC/GMC) [75,76,77] was used for the continuum-level microscale predictions. In the MAC/GMC code, the periodicity of the material microstructure is characterized by generating a repeating unit cell (RUC). The RUC involves a discrete number of subcells, where each subcell can be used to represent a single phase of the composite. There are several built-in architectures (ARCHID) of the RUC; however, the user can define custom architectures. Additionally, the user has the ability to control the fiber volume fraction and aspect ratio within the RUC. The continuum-level modeling steps for prediction of microscale mechanical properties, and the corresponding results, are detailed below. The multiscale modeling workflow is shown in Figure 8, which illustrates the continuum-level steps to design the nanocomposites. It is important to note that other options exist for micromechanics analysis. For example, the Mori-Tanaka approach is commonly used; however, the MAC/GMC approach allows for a more detailed description of the micro-level material topology.

In order to predict the effective mechanical properties of the nanoplatelet/epoxy nanocomposites, a multiscale approach was utilized to incorporate the local interphase properties of the MD models (Table 5, Figure 8a) into the bulk epoxy. The approach involved generating a MAC/GMC RUC (ARCHID = 1) shown in Figure 8b, which contained 8 discretized subcells. One of the subcells was used to incorporate the properties from the MD predictions, and bulk epoxy properties were incorporated in the other seven subcells. The bulk DGEBA-DETDA epoxy properties were taken from the study by Qi et al. [78], in which the experimental elastic modulus was reported to be 2.71 ± 0.11 GPa. Generating this RUC was necessary to control the nanofiller content (vol% and wt%) and its aspect ratio within the epoxy matrix.

For the nanofiller content, it was shown previously [50,54,55] that the overall volume fraction of the GNP, GO, or FGO within epoxy matrix (vol% *_NP/RUC_*) can be calculated using the following expression:

(2) $${\mathrm{vol}\%}_{NP/RUC}={\mathrm{vol}\%}_{NP/MD}\times {\mathrm{vol}\%}_{MD/RUC}$$

where vol% *_NP/MD_* represents the GNP, GO, or FGO volume fraction within the MD model that is given in Table 4, and vol% *_MD/RUC_* represents the volume fraction of the MD model within the RUC that can be adjusted in the MAC/GMC script. A similar approach can be used to specify the nanofiller wt% within the epoxy matrix (wt% *_NP/RUC_*) according to the following expression:

(3) $${\mathrm{wt}\%}_{NP/RUC}={\mathrm{wt}\%}_{NP/MD}\times {\mathrm{wt}\%}_{MD/RUC}$$

where wt% *_NP/MD_* represents the weight percent of GNP, GO, or FGO within the MD model that is given in Table 4, and wt% *_MD/RUC_* represents the weight percent of the MD model within the RUC that can be evaluated using the following expression:

(4) $${\mathrm{wt}\%}_{MD/RUC}=\frac{\rho_{MD}\times {\;\mathrm{vol}}_{MD/RUC\;}}{\rho_{MD}\times {\mathrm{vol}}_{MD/RUC}+\rho_{B}\times {\mathrm{vol}}_{B/RUC}}\times 100\%$$

where $\rho_{MD}$ and $\rho_{B}$ are the mass density of the MD model and bulk epoxy, respectively. The values of $\rho_{MD}$ for the MD models are given in Table 4, while $\rho_{B}$ is ~1.2 g/cm^3^. The bulk epoxy volume fraction within the RUC (vol*_B/RUC_*) can be determined from

(5) $${\mathrm{vol}}_{B/RUC}=1-{\mathrm{vol}}_{MD/RUC}$$

The nanoplatelet aspect ratio ($a_{NP}$) can be controlled using the following expression:

(6) $$a_{NP}=l/t_{NP}$$

where l and $t_{NP}$ are the length and the thickness of the GNP, GO, or FGO nanoplatelets. For the reinforcement subcell in the RUC, the aspect ratio of the nanocomposite MD model associated with the reinforcement subcell ($a_{MD}$) can be calculated using the following expression:

(7) $$a_{MD}=l/t_{MD}$$

where l and $t_{MD}$ are the length and the thickness of the MD model, respectively. According to the RUC settings, the MD model length l is substituted as the reinforcement length while the MD model thickness $t_{MD}$ is substituted as the reinforcement diameter (width). Rearranging Equation (6) for l and substituting into Equation (7) results in the following expression for $a_{MD}$:

(8) $$a_{MD}=\frac{a_{NP}\times t_{NP}}{t_{MD}}$$

The volume fraction of the nanoplatelets in the MD simulation box can be determined with

(9) $$vol_{NP/MD}=\frac{t_{NP}\times l_{2}\times l_{3}}{t_{MD}\times l_{2}\times l_{3}}$$

Because the lateral lengths ($l_{2}$ and $l_{3}$) of the nanoplatelet and the MD simulation box are identical and can be omitted, Equation (8) can be rewritten as

(10) $$a_{MD}=a_{NP}\times {\mathrm{vol}}_{NP/MD}$$

Equations (2)–(10) provide the flexibility to predict the effective mechanical properties of the nanocomposites at different nanofiller contents and aspect ratios without the need for running additional MD simulations. It is important to note that the wrinkle phenomenon becomes more significant as the nanoplatelet aspect ratio increases. Researching this issue is computationally expensive with MD modelling. Fortunately, MAC/GMC allows users to control the nanoplatelets size by adjusting the size of the fiber subcell in the RUC. This helps to extend the effect of the nanoplatelet wrinkling as the aspect ratio increases.

After running the first MAC/GMC script utilizing the RUC settings illustrated in Figure 8b and Equations (2)–(10), the predicted mechanical properties of the nanocomposite were further processed using Christensen and Waals equations [79]. These equations were used to predict the effective homogenized/isotropic mechanical properties of the nanocomposites with randomly orientated nanoplatelets in the epoxy matrix, as shown in Figure 8c. The predictions at this point represent the bulk-scale mechanical properties of the nanoplatelet/epoxy nanocomposites.

## 4. Results and Discussion

The predicted mechanical properties of the nanoplatelet/epoxy nanocomposites are described in this section. Comparison with experimental data from the literature is provided for modeling validation. The nanoplatelet/epoxy composite predictions can be divided into two levels: first, the localized interphase MD predictions, and second, the bulk nanocomposite predictions.

### 4.1. MD Predictions

Referring to Table 5, the predicted elastic properties of the localized interphase regions of GNP/epoxy nanocomposites (Figure 5) using ReaxFF are generally higher than the MD predictions from Hadden et al. [54] using OPLS. A similar trend was observed in a previous work [55] when comparing the predicted mechanical properties of a 4GNP/epoxy using ReaxFF with the predictions from Hadden et al. [54] using OPLS.

In general, introducing the functional and/or oxygen groups to the GNP produced a significant drop in the in-plane elastic ($E_{ip}$) and shear ($G_{ip}$) moduli. Specifically, GO/epoxy exhibits a −89.25% and −74.42% reduction in the $E_{ip}$ and $G_{ip}$, respectively. FGO/epoxy exhibits a −88.94% and −72.43% reduction in the $E_{ip}$ and $G_{ip}$, respectively. A slight drop can be also observed in the out-of-plane modulus ($E_{op}$) with −25.49% for the GO/epoxy and −17.65% for the FGO/epoxy. This drop is principally attributed to the degradation in the robust sp^2^ structure of the GNP due to the functionalization, as shown above in Figure 3. Conversely, surface functionalization produced a tremendous improvement in the out-of-plane shear modulus ($G_{op}$), which increased by 15.45 times for the GO/epoxy and by 19.52 times for the FGO/epoxy. For both systems, this improvement in the $G_{op}$ is attributed partly to the enhancement in the IIE observed between the GO or FGO and epoxy matrix (Figure 7) and partially to the rough and wrinkled surfaces of GO and FGO nanoplatelets (Figure 5), which triggers an interlocking mechanism with the epoxy matrix. For the GFO/epoxy system, the improvement in *G_op_* is also partially due to the additional covalent bonds formed between the nanoplatelet and epoxy matrix. For the Poisson contractions, the in-plane Poisson’s ratio ($\nu_{ip}$) value for the functionalized nanoplatelet composites experiences a significant drop. In contrast, the out-of-plane Poisson’s ratio ($\nu_{op}$) demonstrated a large increase with functionalization. This lateral contraction behavior can be attributed to the alteration in the GNP sp^3^/sp^2^ ratio and nanoplatelet wrinkling.

The results shown in Table 5 indicate that the mechanical properties of the nanocomposite, especially Poisson’s ratio, are highly governed by the nanoplatelet type. This sensitivity of properties to nanoplatelet type is also reported in the literature. Cho et al. [80] stated that the basal plane of the graphite crystal can suffer mobile dislocations; therefore, the elastic properties of graphite vary over a wide range. For example, $\nu_{op}$ was reported to be in the range of −0.171 to 4.958. The mechanical properties of graphene predicted by Jensen et al. [81] (e.g., $E_{ip}$ = 1235 GPa & $\nu_{ip}$ = 0.876, using ReaxFF_CHO_) can be used to justify the current results. Considering the discrepancies between nanoplatelet type and the force field used in the MD modeling and predictions, the predicted mechanical properties in this study show a reasonable agreement with those predicted by Hadden et al. [54]. In fact, using pristine graphene nanoplatelet reinforcement, the mechanical properties predicted for 4GNP/epoxy are more realistic and closer to experiments than those predicted for single layers of GNP/epoxy [54,55].

Generally, the FGO/epoxy exhibits a higher prediction of the localized moduli relative to the GO/epoxy nanocomposite. That is, the nanocomposite localized interphase stiffness is highly governed by the interface structure which asserts the order GNP ≫ FGO > GO.

### 4.2. Nanoplatelet/Epoxy Bulk Predictions

In general, the process of comparing the predicted mechanical properties with experiments involves some challenges. This is due to a wide range of factors that can affect the nanocomposite molecular structure and hence its engineering properties, including the size and number of layers in the graphene nanoplatelets, the exact chemical composition of the resin, residual solvents, and the integrity of the graphene lattice. Addressing all of these factors in MD modeling can be very difficult and time consuming. In this work, the GNP/epoxy system represents an ideal case of pristine and planar single layers of graphene perfectly dispersed in the matrix. Although the GO and FGO nanocomposites involve a different chemical composition of the composite, they are modeled at the same level of dispersion. In a previous study [55], the 4GNP/epoxy was modeled at a specific level of GNP dispersion with varying nanoplatelet volume fractions. The approach used in the current study provides further parameter control. The RUC parameters shown in Figure 8b,c and Equations (2)–(10) enable the prediction of the mechanical properties for bulk nanocomposites at different nanoplatelet volume fractions and aspect ratios.

**Figure 9:**
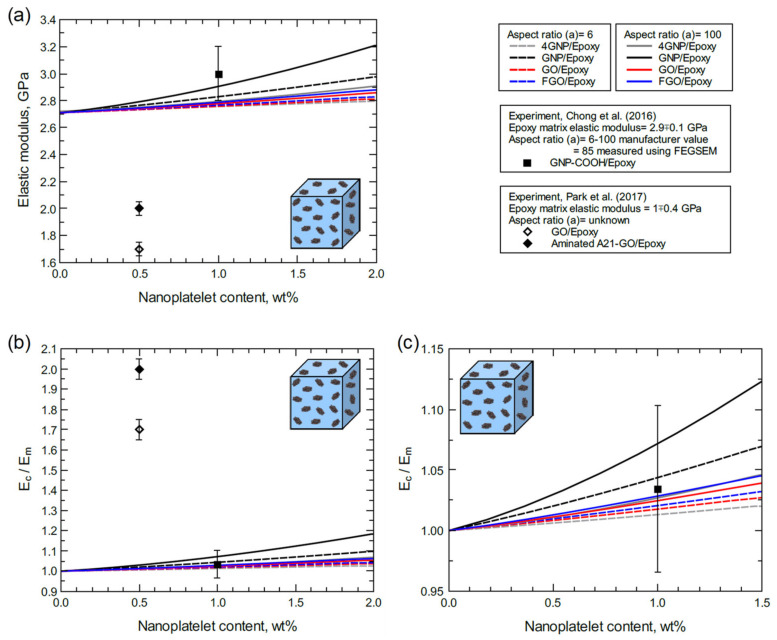
Elastic modulus predicted for various nanoplatelet content levels and compared with experiment, (**a**) unnormalized modulus, (**b**) normalized modulus, and (**c**) focus on GNPCOOH/epoxy modulus at 1.0 wt%.

Figure 9 shows the predicted elastic modulus for each nanocomposite type for different nanoplatelet contents (wt%) at aspect ratios of 6 and 100. The unnormalized predicted moduli (Figure 9a) are significantly higher than the experimental values from Park et al. [34]. The epoxy matrix (DGEBA) modulus in their experiment was reported to be 1 ± 0.4 GPa. This matrix modulus is about two times lower than the typical value reported in the literature [42,78]. In addition, the nanoplatelet aspect ratio is unknown in the experiment. However, the experimental elastic modulus of GNP-COOH/epoxy (and GNP-O_2_/epoxy, which is unreported here as its mechanical properties are identical to the GNP-COOH/epoxy) from Chong et al. [42] indicates a good agreement with the predictions. The normalized composite modulus ($E_{c}$) by the matrix modulus ($E_{m}$) (Figure 9b,c) allows for more realistic validation of the current predictions with the experiment from Chong et al. The experimental aspect ratio of GNP-COOH nanoplatelets measured using field emission scanning electron microscopy (FESEM) was reported to be 85, while the manufacturer value is reported to be 6–100. In general, all the predicted moduli fall within the standard deviation of the experimental modulus from Chong et al. at 1.0 wt% content (Figure 9c). However, the experimental mean value is slightly lower than the predicted moduli for GNP/epoxy composite, which represents the ideal case using intact GNP structures with perfect dispersion in the matrix. The experimental mean value of the modulus is also close the predicted modulus values for 4GNP/, GO/, and FGO/epoxy composites with an aspect ratio of 100, and slightly higher than those with an aspect ratio of 6.

**Figure 10:**
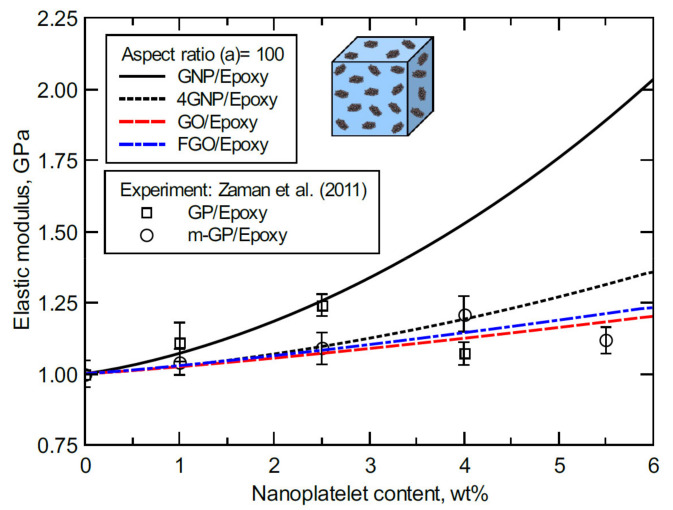
Normalized elastic modulus predicted at 100-aspect ratio for various nanoplatelet contents and compared with experiments from Zaman et al. (2011).

Zaman et al. [27] performed an experimental study to investigate the mechanical response of epoxy reinforced with graphene nanoplatelets (GP) and surface-modified graphene nanoplatelets (m-GP) with carbonyl and amine radical groups attached to the GP surfaces. These reactive functional groups were found to improve the m-GP dispersion and the interfacial strength via formation of covalent bonds between the m-GP and the host matrix. Figure 10 shows the Young’s modulus of GP/epoxy and m-GP/epoxy obtained for various wt% of GP and m-GP content. Clearly, the GP/epoxy samples demonstrated higher Young’s moduli relative to m-GP/epoxy samples as the nanoplatelet content increased from 1.0–2.5 wt%. This not the case at 4.0 wt% of GP and m-GP content since the stiffening effect of GP diminished due to agglomeration. In contrast, the m-GP/epoxy maintained a steady improvement up to 4.0 wt% of m-GP content. A slight deterioration in the obtained Young’s modulus can be observed at 5.5 wt% of m-GP content. Considering the adverse effect of agglomeration on Young’s moduli at higher nanoplatelet contents, the predicted elastic moduli of the nanocomposites modeled herein are in a good agreement with experiment. Specifically, the predicted elastic moduli of GNP/epoxy are in excellent agreement with experimental values obtained at 1.0–2.5 wt% nanoplatelet contents. The experimental Young’s moduli of the m-GP/epoxy at 1.0–2.5 wt% nanoplatelet content are also accurately predicted by the 4GNP/, GO/, and FGO/epoxy nanocomposite models. At higher nanoplatelet contents (4.0–5.5 wt%), a close agreement can be observed between the predicted and experimental moduli. All the moduli predicted herein are for nanoplatelets with a 100-aspect ratio, while the cluster sizes of GP and m-GP at 4 wt% were reported to be 0.7 ± 0.5 µm and 1.8 ± 1.5 µm, respectively. The weak mechanical performance of m-GP/epoxy relative to GP/epoxy at 1.0–2.5 wt% nanoplatelet content supports the current predictions since GO and FGO have weaker a reinforcing effect relative to GNP. This trend does not apply at higher nanoplatelet contents as agglomeration becomes more dominant for unmodified nanoplatelets.

**Figure 11:**
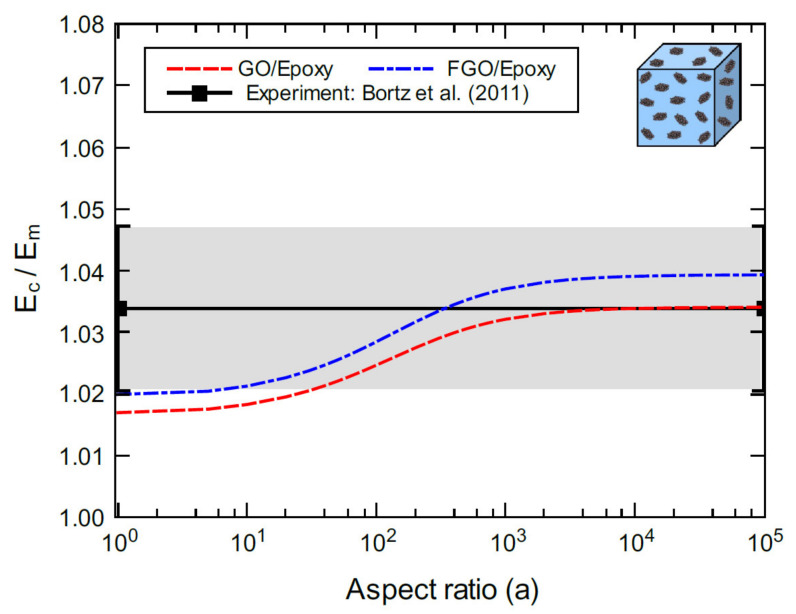
Normalized elastic modulus of GO/epoxy and FGO/epoxy predicted for various aspect ratios at 1.0 wt%. For the experiment, the epoxy matrix modulus is 2.99 ± 0.15 GPa with GO content of 1.0 wt%.

Figure 11 shows the predicted bulk elastic modulus of GO/epoxy and FGO/epoxy nanocomposites ($E_{c}$) normalized by the matrix modulus ($E_{m}$). The moduli are predicted for various aspect ratio values at 1.0 wt% nanoplatelet content. The predictions are in good agreement with the experimental values of the GO/epoxy elastic modulus from Bortz et al. [4]. Even though the GO nanoplatelet dimensions were not identified in the experimental work, the variation in the measured modulus spans a wide range (~10 to infinite) of possible aspect ratios. Figure 11 also indicates an excellent agreement between the mean modulus value from experiment and the predicted modulus of GO/epoxy at aspect ratios spanning between ~10^3^ to infinite. The mean modulus value from experiment and the predicted modulus of FGO/epoxy are in excellent agreement around an aspect ratio of 350.

**Figure 12:**
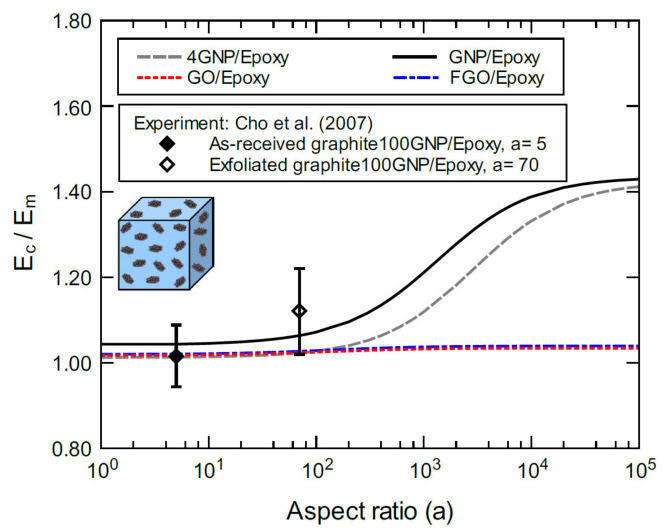
Normalized elastic modulus of 4GNP/, GNP/, GO/, and FGO/epoxy predicted for various aspect ratios at 1.0 wt% nanoplatelet content. For the experiment, the epoxy matrix modulus was 3.27 GPa and the nanoplatelet content was 1.0 wt%.

Figure 12 shows the bulk moduli of the proposed nanocomposites predicted for various aspect ratio values at 1.0 wt% nanoplatelet content compared with experimental data from Cho et al. [80]. The experimental modulus of the as-received graphite 100GNP/epoxy nanocomposite with an aspect ratio of 5 and 1.0 wt% nanoplatelet content is in excellent agreement with the predicted moduli of 4GNP/, GO/, and FGO/epoxy. Additionally, the predicted modulus of GNP/epoxy is slightly higher than the mean value of the experimental modulus, but still within the standard deviation. For the experimental modulus of the exfoliated graphite 100GNP/epoxy nanocomposite with an aspect ratio of 70 and 1.0 wt% nanoplatelet content, the mean value is slightly higher than the predicted modulus. All the predicted moduli, however, are within the lower bound of the standard deviation. It is important to note that the purpose of including GO and FGO nanocomposites in the comparison is to assess their relative performance, which is similar to 4GNP/epoxy for aspect ratios less than 200. In general, these predictions agree with the experiment, which further validates the current modeling method, especially the effect of the particle size of the filler and its role in affecting the properties predicted by the adopted modeling approach.

Among several types of graphene-based nanocomposites tested by Chong et al. [42], the XG-C/epoxy and XG-M/epoxy are used here for validating the modeling approach in this work. The manufacturing process used to prepare the nanocomposites involved using two different types of solvents to disperse the dry powder of graphene nanoplatelets in the matrix. They used ultrasonication with tetrahydrofuran (THF) or n-methyl-pyrrolidone (NMP) solvents that produced two different levels of graphene nanoplatelet dispersion. The NMP solvent produced higher levels of nanoplatelet dispersion relative to the THF. Therefore, the experimental moduli of the nanocomposites using NMP were higher than those using THF. Residual solvent in the nanocomposite is expected to introduce chemical elements that cause chemical alterations to the carbon lattice structure of the graphene, depending on the solvent concentration. In addition, GNP exfoliation and the nanocomposite constituent mixing techniques can greatly affect the nanoplatelet aspect ratio.

**Figure 13:**
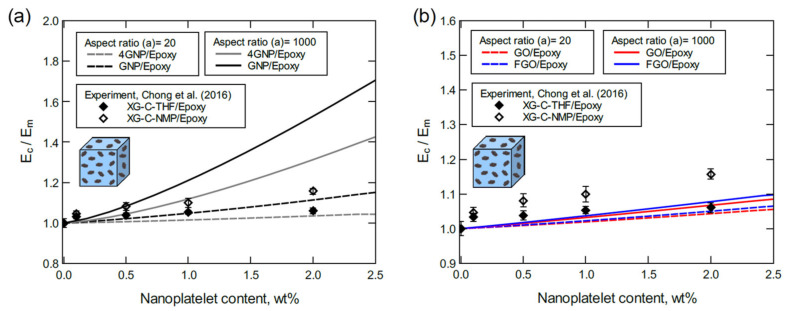
Normalized elastic modulus predicted for various nanoplatelet contents: (**a**) GNP/epoxy and 4GNP/epoxy, (**b**) GO/epoxy and FGO/epoxy. In the experiment, the matrix modulus was 2.9 ± 0.1 GPa, and the aspect ratio was 1000 as the manufacturer value and 19 as measured using FEGSEM.

The predicted moduli from GNP/epoxy and 4GNP/epoxy models shown in Figure 13a are generally in good agreement with experimental moduli of XG-C-THF/epoxy and XG-C-NMP/epoxy systems. In general, as nanoplatelet agglomeration levels increase, the effective aspect ratio decreases because of the general shape of agglomerated clusters. Hence, experimental values of the modulus at low nanoplatelet contents (0.1 and 0.5 wt%) are in excellent agreement with the predicted moduli with the high aspect ratio. However, experimental values of the modulus at higher nanoplatelet contents (1.0 and 2.0 wt%) deviate toward the predicted moduli for the lower aspect ratio. In both cases, the experimental moduli of XG-C-NMP/epoxy are close to the predictions of GNP/epoxy, while the XG-C-THF/epoxy moduli are close to the predictions of 4GNP/epoxy. The experimental moduli were also compared with predicted moduli of the GO/epoxy and FGO/epoxy systems (Figure 13b). Considering the detrimental effect of the high concentration of oxygen in the GO and FGO models, an acceptable agreement with experiment is observed.

**Figure 14:**
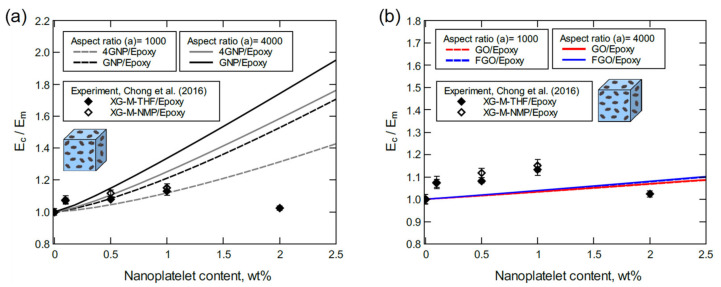
Normalized elastic modulus predicted for various nanoplatelet contents: (**a**) GNP/epoxy and 4GNP/epoxy, (**b**) GO/epoxy and FGO/epoxy. For the experiment, the matrix modulus is 2.9 ± 0.1 GPa, and the aspect ratio is 4167 as the manufacturer value and 1142 as measured using FEGSEM.

To some extent, the judgment used in justifying the results shown in Figure 13 can be also feasible for the predicted moduli compared with experimental moduli of XG-M-THF/epoxy and XG-M-NMP/epoxy shown in Figure 14. However, the large deviation between predicted and experimental moduli at 2.0 wt% of the nanoplatelet content can be attributed to the high agglomeration degree in the experiment.

**Figure 15:**
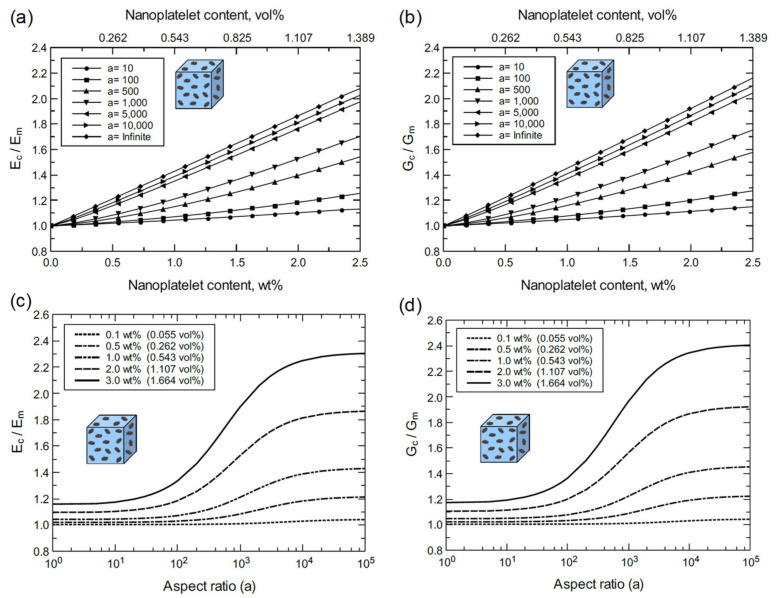
Normalized elastic and shear moduli of bulk GNP/epoxy predicted for various nanoplatelet contents and aspect ratios: (**a**) $E_{c}/E_{m}$ vs GNP content at different aspect ratios, (**b**) $G_{c}/G_{m}$ vs. GNP content at different aspect ratios, (**c**) $E_{c}/E_{m}$ vs. aspect ratio at different GNP contents, and (**d**) $G_{c}/G_{m}$ vs. aspect ratio at different GNP contents.

**Figure 16:**
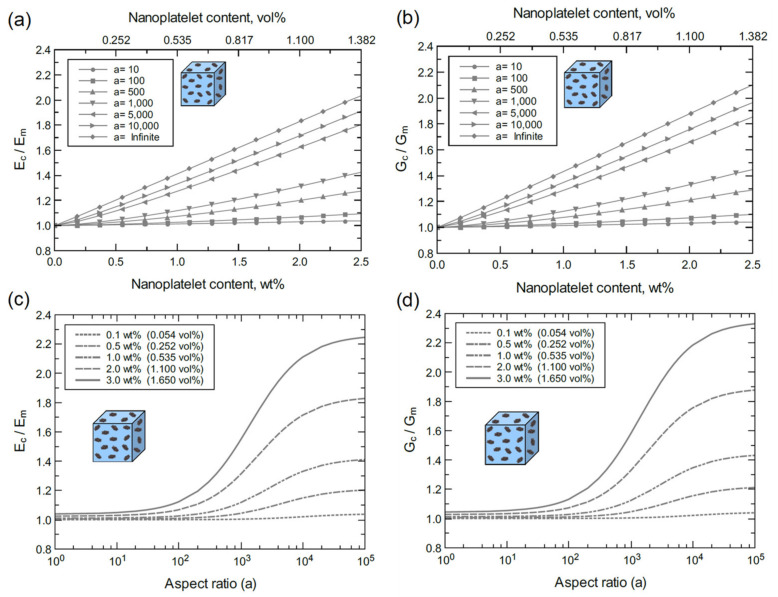
Normalized elastic and shear moduli of bulk 4GNP/epoxy predicted for various nanoplatelet contents and aspect ratios: (**a**) $E_{c}/E_{m}$ vs 4GNP content at different aspect ratios, (**b**) $G_{c}/G_{m}$ vs. 4GNP content at different aspect ratios, (**c**) $E_{c}/E_{m}$ vs. aspect ratio at different 4GNP contents, and (**d**) $G_{c}/G_{m}$ vs. aspect ratio at different 4GNP contents.

The above discussion indicates that the multi-scale modeling approached used herein agrees with experimental findings and is thus experimentally validated. The proceeding discussion is focused on a detailed understanding of the individual effects of nanoplatelet content, aspect ratio, and functionalization on the mechanical properties. For the ideal case with perfect GNP dispersion in the matrix, the elastic ($E_{c}$) and shear ($G_{c}$) moduli of bulk GNP/epoxy are plotted for various GNP contents and aspect ratios (Figure 15). Figure 15a,b show consistent increases in $E_{c}$ and $G_{c}$ with GNP content and aspect ratio. The improvement in $E_{c}$ and $G_{c}$ at 2.5 wt% (1.389 vol%) with an infinite aspect ratio (a >10^4^) is more than 100%. However, the reinforcing effect of GNP plateaus at aspect ratios greater than 10^4^ as indicated in Figure 15c,d. In addition, the reinforcing effect of GNP is limited for aspect ratios below 10^2,^ and it becomes significant within a 10^2^–10^4^ aspect ratio span. This trend can be clearly observed at high GNP contents. At a lower levels of GNP dispersion (4GNP/epoxy), the bulk nanocomposite response exhibits a similar trend to the bulk GNP/epoxy, as shown in Figure 16. However, the reinforcement effectiveness of 4GNP is relatively low due to the agglomeration.

**Figure 17:**
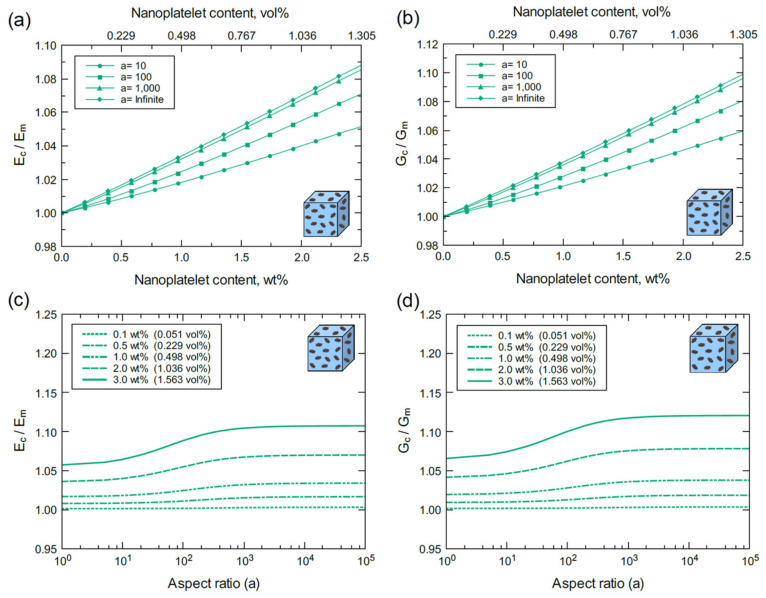
Normalized elastic and shear moduli of bulk GO/epoxy predicted for various nanoplatelet contents and aspect ratios: (**a**) $E_{c}/E_{m}$ vs. GO content at different aspect ratios, (**b**) $G_{c}/G_{m}$ vs. GO content at different aspect ratios, (**c**) $E_{c}/E_{m}$ vs. aspect ratio at different GO contents, and (**d**) $G_{c}/G_{m}$ vs. aspect ratio at different GO contents.

**Figure 18:**
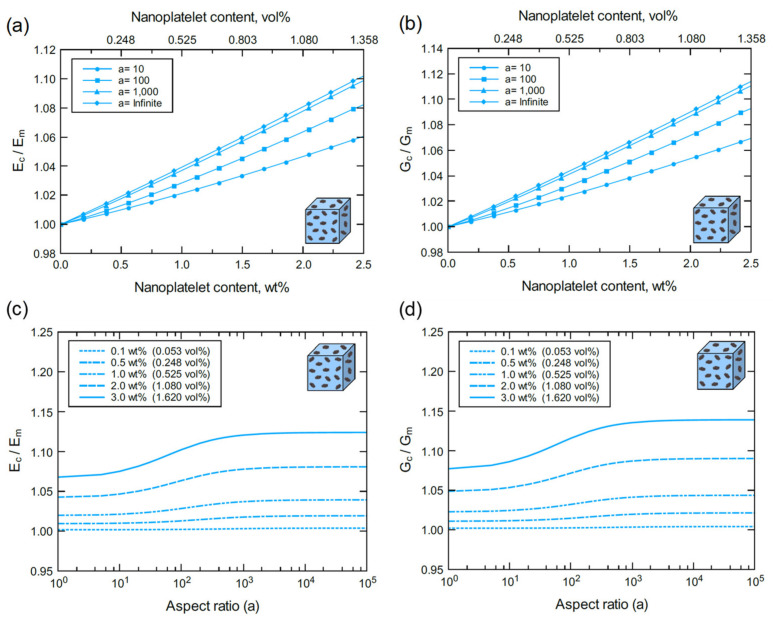
Normalized elastic and shear moduli of bulk FGO/epoxy predicted for various nanoplatelet contents and aspect ratios: (**a**) $E_{c}/E_{m}$ vs. FGO content at different aspect ratios, (**b**) $G_{c}/G_{m}$ vs. FGO content at different aspect ratios, (**c**) $E_{c}/E_{m}$ vs. aspect ratio at different FGO contents, and (**d**) $G_{c}/G_{m}$ vs. aspect ratio at different FGO contents.

Referring to Figure 17 and Figure 18, the mechanical response of bulk GO/epoxy and FGO/epoxy are relatively similar with a slightly better reinforcing function when using FGO nanoplatelets. As discussed above, both GO and FGO have a weaker reinforcing effect in comparison to the GNP. Therefore, the mechanical properties of the GO and FGO nanocomposites at the bulk level involved limited improvement with increasing levels of nanoplatelet content and aspect ratio. Specifically, there is a minor increase in $E_{c}$ and $G_{c}$ with increasing GO and FGO content and aspect ratio. For the GO/epoxy mechanical response shown in Figure 17a,b, the maximum improvement in $E_{c}$ and $G_{c}$ at 2.5 wt% (1.305 vol%) of GO with an infinite aspect ratio (a >10^4^) is ~9% and ~10%, respectively. However, for the FGO/epoxy mechanical response shown in Figure 18a,b, the maximum improvement in $E_{c}$ and $G_{c}$ at 2.5 wt% (1.358 vol%) of FGO with an infinite aspect ratio (a >10^4^) is ~10% and ~11%, respectively. The reinforcing effect of GO and FGO plateaus at aspect ratios greater than 10^3^ as indicated in Figure 17c,d and Figure 18d. The effectiveness of the GO and FGO reinforcement is limited for aspect ratio values below 10^1^ and improves within the 10^1^–10^3^ aspect ratio span. This trend can be clearly observed at high GO or FGO contents.

Considering Figure 15, Figure 16, Figure 17 and Figure 18, it can be generalized that increasing either the nanoplatelet content and/or aspect ratio results in an improvement in the mechanical properties of the nanocomposite. This behavior can be attributed to the increase in the surface contact area between the nanoplatelets and the epoxy matrix that improves the load transfer between them. Therefore, the two key processing parameters of the nanoplatelets (wt% and aspect ratio) provide an integrated reinforcing function to the nanocomposite.

**Figure 19:**
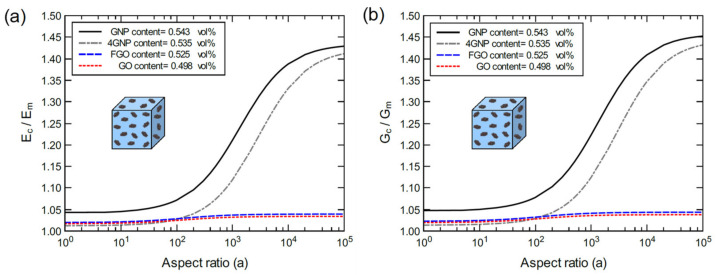
Normalized elastic modulus (**a**) and shear modulus (**b**) for bulk GNP/, 4GNP/, GO/, and FGO/epoxy for various aspect ratios at 1.0 wt% nanoplatelet content.

Figure 19 shows a comparison between the normalized elastic modulus for bulk GNP/, 4GNP/, GO/, and FGO/epoxy for various aspect ratios at 1.0 wt% nanoplatelet content. The volume fraction (vol%) for each nanoplatelet is also provided. The discrepancy between wt% and vol% can be attributed to the number of atoms, chemical composition, and wrinkled morphology in each case. Clearly, the best response can be observed for the bulk GNP/epoxy nanocomposite, which has the highest dispersion level of GNP. The bulk 4GNP/epoxy exhibits a consistent lower response relative to the bulk GNP/epoxy, which is governed by the lower dispersion level in the 4GNP. However, the difference reduces at aspect ratio values larger than 10^4^. Both the GO/epoxy and FGO/epoxy systems exhibit a nearly identical response with the FGO/epoxy slightly higher. For those systems, increasing the aspect ratio results in a very small improvement in the mechanical response. This can be mainly attributed to the detrimental effect of the high concentration of oxygen introduced to the GNP as discussed above. The mechanical response of the GO and FGO systems is comparable with the bulk 4GNP/epoxy response at aspect ratio values below 10^2^. The results indicate that the GNP/epoxy composite response can be assumed to be the ideal case or the upper bound of the mechanical response. The FGO/epoxy or GO/epoxy systems can be assumed to be the lower bound of the mechanical response. That is, GNP nanoplatelets with lower oxygen concentrations are expected to exhibit an intermediate mechanical response.

Regardless of the broad goal, which is to reduce the time and effort to design and manufacture epoxy-based nanocomposite materials using computational modeling, this work provides important information about using functionalized graphene nanoplatelets to reinforce epoxy matrices. The main advantages of using functional groups in GO and FGO is the increase in the interfacial binding and the nanoplatelet wrinkling and surface roughness, which improves the mechanical interlocking between the nanoplatelets and the epoxy matrix. In addition, the process of surface functionalization of the nanoplatelets helps to increase the interlayer spacing between graphene nanosheets in the graphene nanoparticles. This has the effect to improve and maintain the dispersion of the nanoplatelets for two reasons. First, functionalization reduces the nanoplatelet restacking as the pi-pi stacking effect is much lower than with pristine nanoplatelets. Second, interfacial binding between the nanoplatelets and the epoxy matrix helps to preserve the nanoplatelet dispersion level. However, there is a tradeoff effect of nanoplatelets surface functionalization since excessive functional groups degrade the nanoplatelet strength, which in turn limits its reinforcing function.

## 5. Summary and Conclusions

A multiscale computational study has been performed to assess the mechanical performance of epoxy composites reinforced with functionalized graphene nanoplatelets. A single layer of GNP was initially modeled using MD to represent the pristine nanoplatelet of graphene and then modified to create a highly concentrated graphene oxide (GO) nanoplatelet and functionalized graphene oxide (FGO) nanoplatelet. The three nanoplatelets were used to reinforce the epoxy matrix (DGEBA-DETDA epoxy system) and generate three MD models of nanoplatelet/epoxy nanocomposites. These MD models were used to predict the localized mechanical properties of the interphase region and to address the interaction energy between each of the nanoplatelets and the hosting matrix. The outcome at the nanoscale indicated a significant degradation in the reinforcing effect of the GO and FGO systems relative to the pristine GNP system. The weakness in the mechanical performance of GO and FGO originates from the alteration in the carbon lattice structure of GNP from its robust sp^2^ structure to the more compliant sp^3^ structure. As a result, FGO/epoxy and GO/epoxy MD models demonstrate a less stiff mechanical response relative to GNP/epoxy. For instance, introducing oxygen groups to the GNP resulted in in-plane elastic and shear moduli of the GO/epoxy composite less than that for the GNP/epoxy system by 89.25% and 72.42%, respectively. On the other hand, a drastic improvement in the interfacial interaction energy was observed when introducing functional and/or oxygen groups to the GNP. More specifically, the interfacial interaction energy of the FGO/epoxy system surpassed that for GO/epoxy and GNP/epoxy by 113.6% and 573.6%, respectively. The surface roughness and wrinkled morphology of the GO and FGO systems along with high interfacial interaction energy were found to improve the interlocking mechanism with the hosting matrix.

The predicted mechanical properties at the nanoscale level were then further analyzed using a micromechanics approach to predict the mechanical response of the nanocomposites at the bulk level for various nanoplatelet content and aspect ratio values. Despite the diversity in the factors, which could potentially affect the mechanical response of the nanocomposites, such as the nanoplatelet dispersion/agglomeration, content, aspect ratio, and their chemical composition, the predictions were in good agreement with experimental results available from the literature. At the bulk level, the optimal mechanical response was observed for the bulk GNP/epoxy nanocomposite. This can be attributed to the perfect dispersion of the strong GNP within the hosting matrix. Thus, its mechanical response can be assumed as the upper bound limit. However, the mechanical response of GO/epoxy and FGO/epoxy can be assumed as the lower bound limit. Any other cases of GNP with oxygen concentration lower than that used herein are expected to register a mechanical response between the designated upper and lower bounds. The predictions indicate that the bulk GO/epoxy and FGO/epoxy can produce a comparable mechanical response to the bulk GNP/epoxy at low nanoplatelet content and aspect ratio values. At larger nanoplatelet contents and aspect ratios, however, the detrimental effect of the oxygen and functional groups on the GNP integrity dominates the overall mechanical response.

## Figures and Tables

**Table 1:** Elemental content (at%) and atomic data used to model the nanoplatelets.

Nanoplatelet			GNP	GO	FGO
Elemental contents (at%) [34]	core-level spectra, Cls		---	60.65	63.98
	core-level spectra, Ols		---	39.35	31.67
	core-level spectra, Nls		---	---	4.35
Number of atoms used in MD modeling	C atoms in the GNP lattice		836	836	825
	Oxygen groups	Epoxide: –O–	---	271 × 1	207 × 1
		Hydroxyl: –OH	---	271 × 2	207 × 2
	Nitrogen groups content	Amine: –NH_2_	---	---	34 × 3
		Amide: –(O=C–NH_2_)	---	---	12 × 5
		Graphitic Nitrogen: –N–	---	---	11 × 1
	Total number of atoms		836	1649	1619
	sp^3^/sp^2^ ratio for the GNP lattice		---	0.973	0.798
	C:O ratio in the nanoplatelet		---	1.542	1.936

**Table 2:** The distance between stacked platelets (d-spacing) in GNP, GO, and FGO.

d-Spacing in (Å)			Reference
GNP	GO	FGO	
3.30	10.86	11.4	Current prediction using box size approach
3.31	9.50	9.56	Current prediction using center of mass approach
3.40	8.59	7.69	Experimental, by Park et al. [34]
3.40	---	8.20	Experimental, by Xiao et al. [31]
3.34	8.30	11.0	Experimental, by Navaee and Salimi [32]
---	6–12	---	Experimental, by Buchsteiner et al. [69]

**Table 3:** Waviness factor of the nanoplatelets.

Nanocomposite MD Model	$\mathrm{Waviness}\;\mathrm{Factor}\;(\mathrm{WF})=L^{w}/L^{a}$			
	GO/Epoxy		FGO/Epoxy	
	WF_*x*_	WF*_y_*	WF*_x_*	WF*_y_*
Model_01	0.875	0.880	0.956	0.915
Model_02	0.954	0.913	0.902	0.874
Model_03	0.889	0.918	0.884	0.911
Model_04	0.896	0.900	0.918	0.874
Model_05	0.872	0.934	0.903	0.906
Average	0.897	0.909	0.912	0.896
Overall WF	0.903		0.904	

**Table 4:** The GNP, GO, and FGO content within their nanocomposite MD models, and the mass density of each MD model.

MD Model	GNP/Epoxy	GO/Epoxy	FGO/Epoxy
Total number of atoms	7028	7841	7811
Mass density (ρ), g/cm^3^	1.27 ± 0.01	1.42 ± 0.01	1.38 ± 0.01
Nanoplatelet content (wt%)	19.58	31.53	29.52
Nanoplatelet content (vol%)	11.36 ± 0.09	19.16 ± 0.24	18.15 ± 0.17

**Table 5:** The MD predictions of the localized interphase region effective mechanical properties.

Mechanical Properties	GNP/Epoxy	GO/Epoxy	FGO/Epoxy	GNP/Epoxy [54]	4GNP/Epoxy [55]
In-plane elastic modulus ($E_{ip}$), GPa	127.5 ± 1.6	13.7 ± 2.3	14.1 ± 2.2	94.10	420.5 ± 2.5
Out-of-plane elastic modulus ($E_{op}$), GPa	5.1 ± 0.5	3.8 ± 0.9	4.2 ± 0.5	2.432	5.3 ± 0.6
In-plane shear modulus ($G_{ip}$), GPa	30.1 ± 0.9	7.7 ± 1.1	8.3 ± 0.8	2.430	102.0 ± 1.0
Out-of-plane shear modulus ($G_{op}$), GPa	0.073 ± 0.021	1.201 ± 0.214	1.498 ± 0.239	0.001	0.019 ± 0.007
In-plane Poisson’s ratio ($\nu_{ip}$)	0.964 ± 0.003	0.080 ± 0.021	0.071 ± 0.043	0.144	0.993 ± 0.001
Out-of-plane Poisson’s ratio ($\nu_{op}$)	0.020 ± 0.007	0.321 ± 0.067	0.267 ± 0.032	0.290	0.002 ± 0.001

## Data Availability

The data presented in this study is available on request from the corresponding author. The data is not publicly available because it is currently being used for further studies.
